# Retiform hemangioendothelioma of the breast: a case report and considerations for effective management

**DOI:** 10.1093/jscr/rjag705

**Published:** 2026-08-21

**Authors:** Georgios E Papanikolaou, Stamatios A Merkouris, Kassiani P Giannaki, Zoi E Papaefthymiou, Stergios E Douvetzemis

**Affiliations:** 4^th^ Breast Clinic, Metropolitan General Hospital, 264 Mesogion Avenue, Cholargos, Athens 15562, Greece; Breast Center, Metropolitan General Hospital, 264 Mesogion Avenue, Cholargos, Athens 15562, Greece; Prolipsis Breast Center, Sevasteias 1 & Michalakopoulou 88A, Ilisia, Athens 11527, Greece; 4^th^ Breast Clinic, Metropolitan General Hospital, 264 Mesogion Avenue, Cholargos, Athens 15562, Greece; 4^th^ Breast Clinic, Metropolitan General Hospital, 264 Mesogion Avenue, Cholargos, Athens 15562, Greece; Department of Basic and Clinical Sciences, Medical School, University of Nicosia, 93 Agiou Nikolaou Street, Engomi, Nicosia 2408, Cyprus

**Keywords:** breast, retiform hemangioendothelioma, vascular neoplasm, intermediate malignancy

## Abstract

Retiform hemangioendothelioma (RHE) is a rare vascular neoplasm of intermediate malignancy, with a high frequency of local recurrence and negligible metastasizing potential. We describe a 45-year-old female who presented with a firm subcutaneous nodule in the lower-outer quadrant of the right breast. Diagnosis of RHE was confirmed based on the characteristic retiform pattern of the branching neoplastic vascular channels, as well as positivity for CD31 and negativity for D2-40 markers. The patient was successfully treated with wide local excision of the tumor with clear surgical margins. The resulting defect was reconstructed with local tissue displacement and direct closure. RHE must be considered in the differential diagnosis of skin vascular tumors, while histopathological and immunohistochemical examination remain core to the diagnosis. Wide local excision with clear margins is the treatment of choice, whereas radiotherapy and, more rarely, chemotherapy can be considered in the event of recurrence and in unresectable cases.

## Introduction

Retiform hemangioendothelioma (RHE) is a rare vascular neoplasm of intermediate malignancy, characterized by locally aggressive and rarely metastasizing potential [1]. Currently, only about 50 cases of RHE have been reported, affecting mainly adolescents and young adults, with a predilection for females [2, 3].

The lesion is most often located in the skin and subcutaneous tissue of the distal lower extremities and occasionally on the head and trunk [2]. Clinically, RHE is presented usually as a single, slowly enlarging, poorly defined, large and indurated nodular or plaque-like lesion, involving the dermis and subcutaneous tissue [3].

We herein report a rare case of a female patient diagnosed with RHE in the skin of the breast and treated successfully with wide local excision. Since the breast is an uncommon site of involvement and scientific evidence is limited, we aimed to highlight the importance of a detailed diagnostic approach and the necessity of an effective treatment with long-term follow-up, proposing recommendations for the management of cases with RHE.

## Case report

A 45-year-old female patient presented with a palpable mass in the lower-outer quadrant of her right breast, without any accompanying symptoms. Ηer family history was not indicative of hereditary breast cancer. Physical examination revealed the presence of a 1-cm, discrete, well-defined, and mobile subcutaneous nodule at the lower-outer quadrant of the right breast, exactly on the inframammary fold. There was no clinical axillary lymphadenopathy.

**Figure 1:**
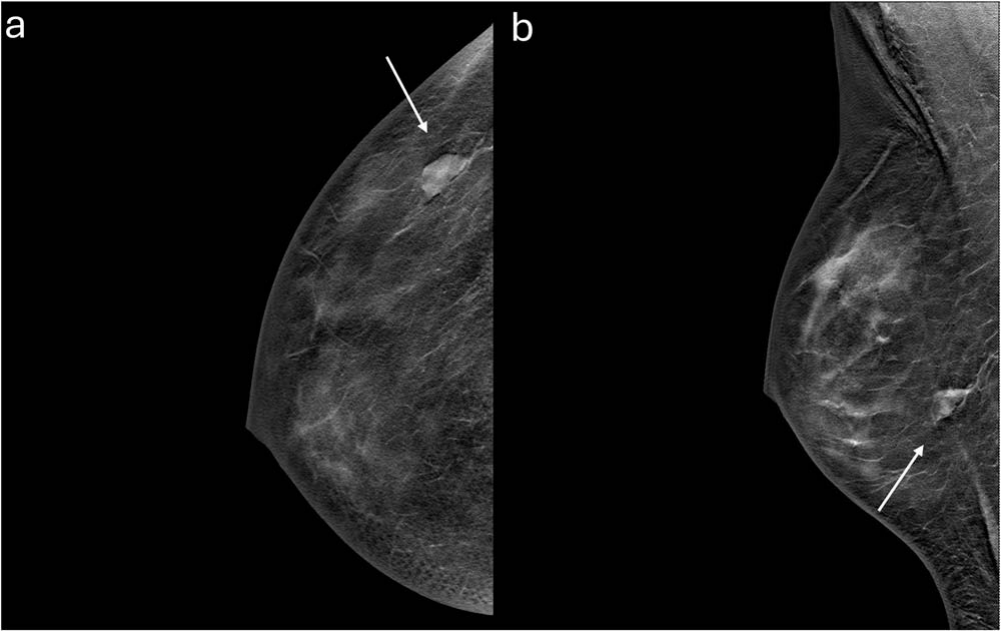
Digital mammography of the right breast (a, craniocaudal and b, lateromedial oblique views) showing a well-circumscribed nodular density without calcifications (arrows).

**Figure 2:**
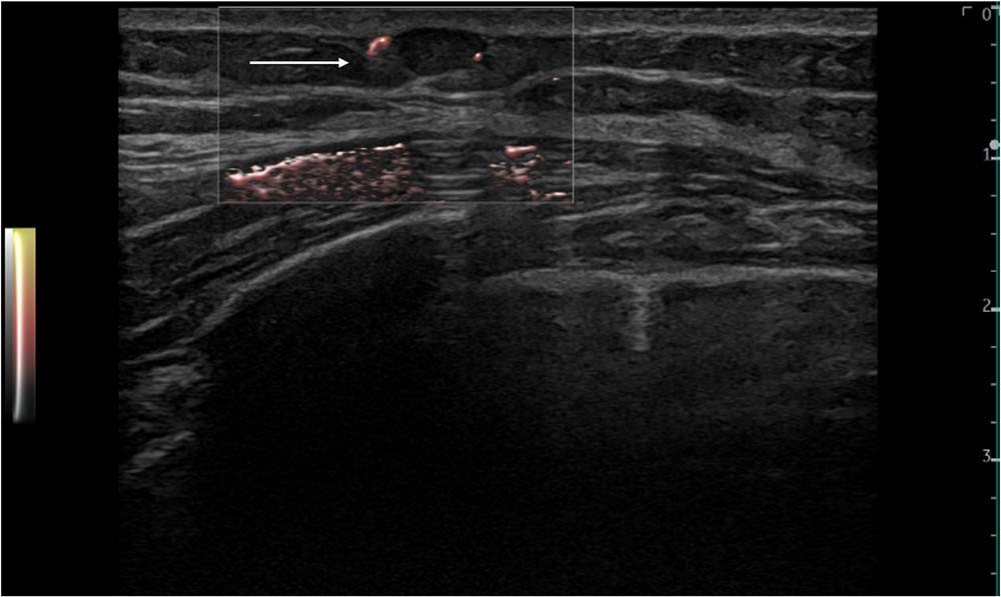
The color-Doppler ultrasonography of the breast revealed a subcutaneous, fairly well-defined, hypoechoic lobular lesion, with posterior acoustic enhancement, measuring 7.5 x 6.5 x 4.5 mm with low internal blood flow (arrow).

The patient was sent for bilateral tomosynthesis and targeted right breast ultrasound. On tomosynthesis, the lesion in the right lower-outer quadrant was well-circumscribed, without calcifications, measuring 13 × 7 mm (Fig. 1a and b), while on ultrasound it was fairly well-defined, hypoechoic, lobulated with low internal blood flow, measuring 7.5 × 6.5 × 4.5 mm and located superficially in the subcutaneous tissue (Fig. 2). There was no evidence of abnormal axillary lymph nodes.

During the ultrasound scan, a core biopsy of the finding was performed. Histology showed irregularly arborizing and dense vascular channels lined by neoplastic endothelial cells with mild nuclear hyperchromasia. Immunohistochemically, the endothelial cells were positive for CD31, CD34, and ETS-related gene, and negative for cellular-MYC.

**Figure 3:**
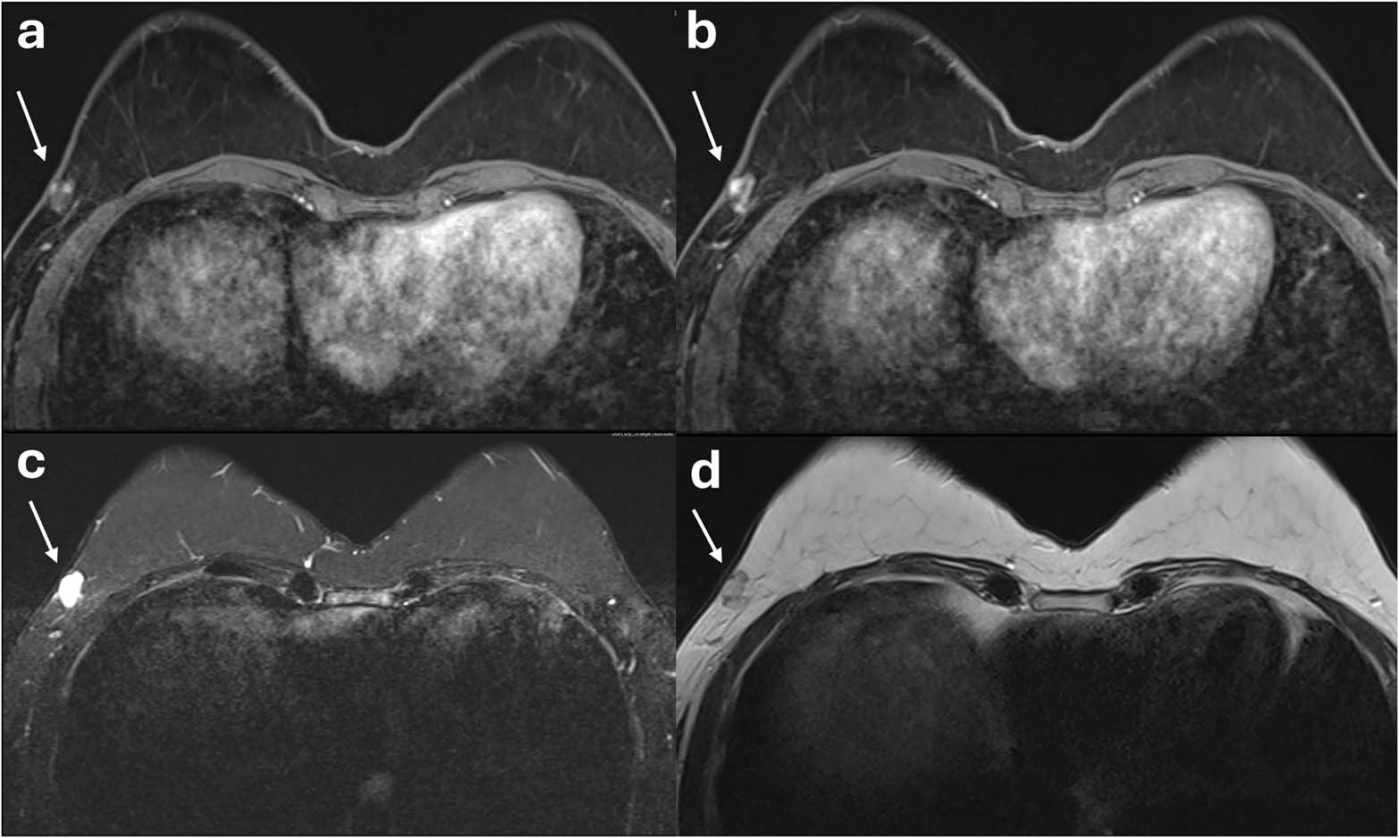
**(**a) T1-weighted MRI in the early phase revealed a 15 × 8 mm lesion in the lower lateral quadrant of the right breast (arrow), (b) T1-weighted MRI in the late phase with a gradual contrast accumulation (arrow), (c) STIR MRI sequence showing a very high signal intensity of the mass (arrow), (d) T2-weighted MRI sequence showing an intermediate signal intensity throughout the lesion in the lower lateral part of the right breast (arrow).

Subsequently, the patient underwent contrast-enhanced magnetic resonance imaging (MRI) of the breast. The T1-weighted MRI revealed a 15 × 8 mm lesion in the lower lateral quadrant of the right breast with an early contrast accumulation and a gradual contrast accumulation in the late phase (Fig. 3a and b). The Short Tau Inversion Recovery MRI sequence showed a very high signal intensity, involving the soft tissues of the lower-outer quadrant of the right breast (Fig. 3c). However, the intermediate signal intensity in the T2-weighted MRI sequence raised the suspicion of a tumor (Fig. 3d).

Since the diagnostic work-up was indicative of a malignant vascular lesion, we proceeded with wide local excision of the tumor, including part of the underlying anterior serratus muscle, because of the proximity of the neoplasm to the muscle. The aim was to achieve a 3-cm wide, tumor-free margin. No axillary surgery was performed; however, during the resection, a palpable lymph node, located next to the tumor, was identified and was included in the excision. Intraoperative digital mammography, as well as frozen section biopsy confirmed the presence of tumor-free margins on the resected specimen. The resulting defect was covered with local tissue displacement, and the skin was closed directly. The postoperative course was uneventful.

**Figure 4:**
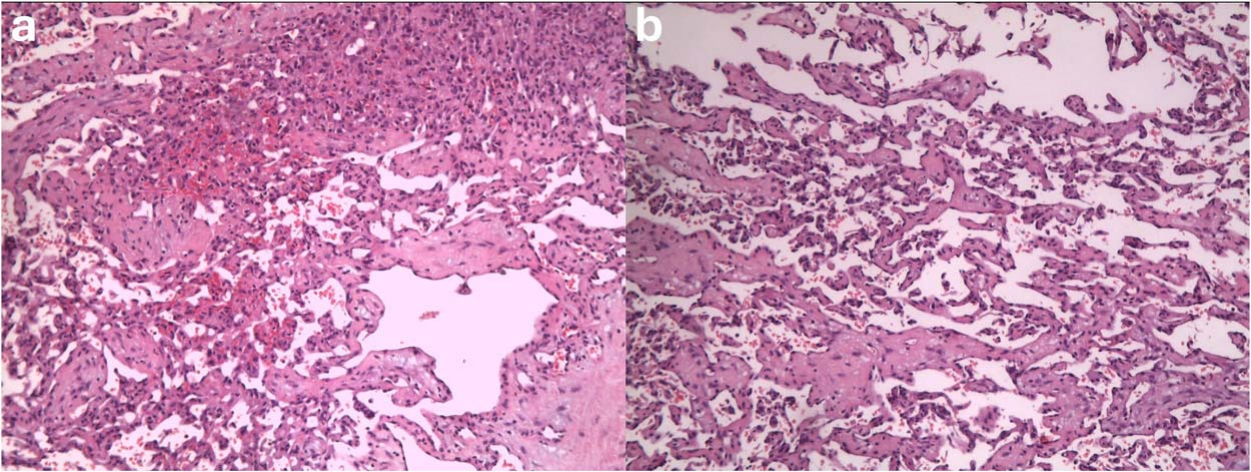
(a) Histological section of the tumor showing the presence of elongated anastomosing vascular channels arranged in retiform pattern (hematoxylin & eosin, magnification ×100), (b) the neoplastic vessels are lined by a single layer of protruding, monomorphic, and non-mitogenic hobnailed endothelial cells (hematoxylin & eosin, magnification ×100).

**Figure 5:**
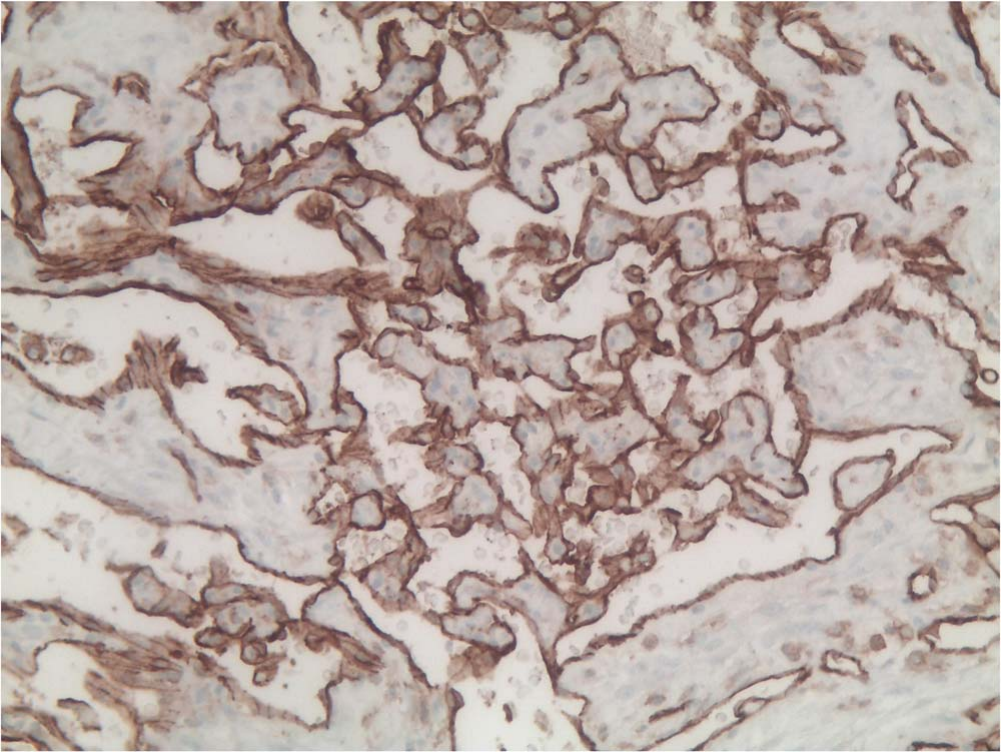
Immunohistochemistry image showing strong positive expression for CD31 of the tumor endothelial cells (magnification ×200).

The histopathologic examination showed the presence of elongated anastomosing vascular channels located in the subcutaneous tissue, lined by protruding, monomorphic, and non-mitogenic hobnailed endothelial cells (Fig. 4a and b). The neoplastic cells expressed the CD31 marker, while D2-40 was negative (Fig. 5). The margins of the resected specimen were tumor-free, and the excised part of the muscle and lymph node were also negative. The final diagnosis was RHE. The patient didn’t show any local recurrence after 12 months of close clinical and radiological follow-up.

## Discussion

The present is an extremely rare case of RHE, presenting as a suspicious, single nodular lesion, located superficially in the subcutaneous tissue of the breast in a female patient. There was no infiltration of the mammary gland, the regional lymphatic and muscular tissues. To the best of our knowledge, only two similar cases of breast RHE have been reported to date, one of them involving the breast gland and the other the subcutaneous tissue [4, 5]. Most of the reported studies are limited to case reports and series, where RHE is mainly located on the head and lower extremities [2, 6–8].

Given the rarity of this neoplasm, the diagnostic approach could be challenging. The differential diagnosis requires high clinical suspicion and detailed imaging and histopathologic examination. RHE must be differentiated from similar cutaneous vascular neoplasms with intermediate biological behavior between the benign hemangioma and malignant angiosarcoma [3, 8].

The therapeutic approach for our patient included a wide local excision of the lesion with negative tumor margins. The decision for a 3-cm wide margin was based on the initial core biopsy histology that raised the suspicion of a malignant vascular lesion and specifically primary angiosarcoma of the breast, in accordance with the prevailing international consensus. Since the neoplasm was located at the level of the inframammary fold and in the vicinity of the underlying thoracic wall, we also proceeded with resection of part of the anterior serratus muscle. The resulting defect was covered with local tissue rearrangement and direct closure. Although the surgical approach remains the primary treatment option, the use of Mohs micrographic surgery has been successfully reported in cases of cutaneous RHE [9, 10]. Radiation therapy has been administered, occasionally in combination with chemotherapy, to patients with unresectable RHE in critical anatomic sites (i.e. head and neck), achieving regression of the lesion [11, 12]. However, radiotherapy is mainly indicated in cases of recurrence after surgical excision and in the presence of lymph node metastases [2, 6, 13]. Immunotherapy with recombinant interferon alpha has been also used along with surgery and radiotherapy in a patient with recurrent RHE of the abdominal wall [14]. Conservative treatment with a combination of pulsed dye laser, local corticosteroid injection, and application of imiquimod cream has been reported in a case of cutaneous RHE on the face with partial response and successful local control of the disease [15].

After 12 months of follow-up, the patient is disease free and with no signs of local recurrence. We emphasize the importance of close and long-term follow-up including clinical examination every 3 months for the first 2 years, breast ultrasound every 6 months for the first 5 years, and an annual mammogram with digital breast tomosynthesis, since local recurrence can be seen many years after the diagnosis of RHE and in ~60% of the cases [2, 8, 15].
